# Application of data mining for predicting hemodynamics instability during pheochromocytoma surgery

**DOI:** 10.1186/s12911-020-01180-4

**Published:** 2020-07-20

**Authors:** Yueyang Zhao, Li Fang, Lei Cui, Song Bai

**Affiliations:** 1 Library of Shengjing Hospital of China Medical University, Shenyang, 110004 China; 2 Department of Information Management and Information System (Medicine), China Medical University, Shenyang, 110001 China; 3 Department of Urology, Shengjing Hospital of China Medical University, 36 Sanhao Street, Shenyang, 110004 Liaoning China

**Keywords:** Data mining, Pheochromocytoma, Relief-F, Naive Bayes, Decision trees, Random forest, Logistic regression

## Abstract

**Background:**

Surgical resection of pheochromocytoma may lead to high risk factors for intraoperative hemodynamic instability (IHD), which can be life-threatening. This study aimed to investigate the risk factors that could predict IHD during pheochromocytoma surgery by data mining.

**Method:**

Relief-F was used to select the most important features. The accuracies of seven data mining models (CART, C4.5, C5.0, and C5.0 boosted), random forest algorithm, Naive Bayes and logistic regression were compared, the cross-validation, hold-out, and bootstrap methods were used in the validation phase. The accuracy of these models was calculated independently by dividing the training and the test sets. Receiver-Operating Characteristic curves were used to obtain the area under curve (AUC).

**Result:**

Random forest had the highest AUC and accuracy values of 0.8636 and 0.8509, respectively. Then, we improved the random forest algorithm according to the classification of imbalanced data. Improved random forest model had the highest specificity and precision among all algorithms, including relatively higher sensitivity (recall) and the highest f1-score integrating recall and precision. The important attributes were body mass index, mean age, 24 h urine vanillylmandelic acid/upper normal limit value, tumor size and enhanced computed tomography difference.

**Conclusions:**

The improved random forest algorithm may be useful in predicting IHD risk factors in pheochromocytoma surgery. Data mining technologies are being increasingly applied in clinical and medical decision-making, and provide continually expanding support for the diagnosis, treatment, and prevention of various diseases.

## Background

Pheochromocytoma is a rare neuroendocrine tumor and the primary treatment strategy is surgical resection; however, the surgery may result in a life-threatening situation with high risk of intraoperative instability of hemodynamics (IHD) due to the excessive release of catecholamine (CA) into the blood circulation [1]. Some independent risk factors possibly related with IHD were identified by statistical methods in previous studies, including tumor size, CA level, preoperative blood pressure, and surgical approaches [2, 3].

Data mining is defined as analyzing observation datasets (generally large-scale datasets) to identify unexpected relationships and summarize the data in a novel pattern, and then provide useful information [4]. Data mining algorithms are classified into two functional types, predictive and descriptive [5], and eight application types, classification, estimation, prediction, correlation analysis, sequence, time sequence, description, and visualization [6]. The successful application of data mining in biomedical research provides reliable support for clinical decision-making (e.g., disease diagnosis, therapy selection, and disease prognosis prediction) and management decision-making (e.g., staffing, medical insurance, and quality control) [7–21].

Although there have been significant improvements in the preoperative medical preparation (PMP), anesthetization, and surgical techniques for pheochromocytoma in recent years, exploring the risk predictors of IHD will bring better therapeutic results. Unfortunately, only a few small-scale retrospective studies have focused on the relevant issues and reached different conclusions, thus the risk factors remain unknown. However, compared with traditional statistical models, data mining can provide better classification results. This study uses data mining to investigate the risk factors that could predict IHD during pheochromocytoma surgery and provides a basis for optimizing patient preoperative preparation and facilitating clinical treatment.

### Pheochromocytoma

Pheochromocytoma is a rare neuroendocrine tumor that originates from the adrenal medulla chromaffin cells and can secrete one or more CAs, including epinephrine, norepinephrine, and dopamine. The incidence rate of pheochromocytoma is 0.2–0.8/100,000 annually and is 0.1–1% in patients with hypertension. At present, about 25% cases of pheochromocytoma are incidentally found by imageological examination, and pheochromocytoma occurs in 4–5% of patients with adrenal incidentaloma [22]. In addition, pheochromocytoma can cause a series of clinical symptoms due to excessive CA production, including hypertension, headache, sweating, palpitation, tremor, and facial pallor. These symptoms are usually paroxysmal and may be spontaneous or caused by such events as intense physical activity, childbirth, trauma, anesthetic induction, and surgery [23].

Although surgical resection is the major treatment strategy for pheochromocytoma, the surgery is associated with a high risk of intraoperative instability of hemodynamics (IHD) due to excessive release of CA into the blood circulation, which may result in life-threatening conditions [1]. The mortality rate of pheochromocytoma can be as high as 50% during the period when no α-receptor blocker is used to control blood pressure before operation; developments of anesthesiology and surgery and improvements in the pathophysiological comprehension of pheochromocytoma have significantly reduced operative mortality rate to 0–2.9% [23]. However, pheochromocytoma surgery still has high technical requirements and high risk, and thus needs careful PMP.

High fluctuation of blood pressure (hypertension or hypotension), tachycardia or bradycardia, and other IHD manifestations are common during pheochromocytoma surgery, and the “rollercoaster-type” blood pressure is a highly alerted event for surgeons and anesthesiologists. Such IHD may lead to increased intraoperative bleeding and cardio-cerebro-vascular incidents, resulting in increased surgical difficulty and risk. Therefore, to reduce occurrence of IHD and decrease its frequency and amplitude, it is very important to study and determine the risk factors of IHD.

In previous retrospective studies, the influential factors of IHD were predicted. For instance, tumor size was considered to be associated with occurrence of IHD [1, 24, 25]; statistical analysis showed that urinary norepinephrine was a risk factor for IHD [2, 3]; and some other risk factors were also mentioned, such as urine CA, diabetes/prediabetes, large preoperative systolic blood pressure fluctuation, CA level, preoperative blood pressure, and surgical approaches. Few studies have addressed the risk factors of IHD during pheochromocytoma surgery, mainly because the number of cases is small and case collection is difficult. The number of cases collected this time was the highest among all published studies.

### Data mining in healthcare and biomedicine

With the continuous increase in medical big data (containing a lot of patient, disease, surgical, and drug information), it is absolutely necessary to extract potential information about the diagnosis, treatment, and prognosis of diseases and the medical treatment through analysis and knowledge digging. Therefore, the data mining field is closely related to the biomedical field. Many scholars have successfully used data mining to diagnose diseases, predict disease prognosis, and provide decision-making support in the medical field.

Shukla et al. [7] determined the survival rate of breast cancer and predicted its relevant factors by combining self-organizing map with density-based spatial clustering of applications with noise. Their analysis could also help decision-makers to select the best survival period and thus obtain better accuracy of survival prediction. Using regression analysis, artificial neural network, and Naive Bayes, Sangi et al. [8] established a diabetes prediction model to indicate the relationship between the risk factors and the complications in each patient, which could help patients change their lifestyle and implement effective interventions. Moreover, Umesh and Ramachandra [9] explored the feasibility of association rule mining for predicting recurrence of breast cancer in the SEER breast cancer patient database. In a study of disease diagnosis, Akben [10] proposed an automatic diagnosis method for chronic kidney disease; Mostafa et al. [11] extracted the feature set of the human voice and applied five classification algorithms to analyze speech disorders and improve diagnosis of Parkinson’s disease; and Bang et al. [12] established a four-phase data mining model consisting of four modules to select the important diagnostic criteria for effective diagnosis and to predict and diagnose senile dementia. In the field of medical decision-making support, data mining can help third-party payers (e.g., health insurance organizations) extract useful knowledge from thousands of claims and identify a small number of claims and claimants for further evaluation and review of insurance fraud and abuse [13]. Bosson-Rieutort et al. [14] analyzed non-Hodgkin’s lymphoma in the National Occupational Disease Surveillance and Prevention Network Database of France using spectroscopy, and identified 40 occupational exposures related to diseases – this contributed to the monitoring and assessment of occupational exposures associated with health risks. In addition, Chang V et al. used uplift modeling to predict the patient appropriate for ambulatory cleft repair. The uplift modeling is a predictive analytics technique, which was utilized using multivariate logistic regressions [26].. Kartoun U et al. developed an insomnia classification algorithm to identify insomnia patients, and the algorithm had better performance compared with traditional methods [27].

Many machine learning methods have been used for medical data classification and disease factor analysis, and many innovative research results have been achieved. However, due to the inherent high-dimensional feature space, high feature redundancy, and imbalance of sample types in medical clinical data and microarray expression data, especially due to the many factors affecting disease or the complex interactions between genes, the inter-correlations are very strong, and the classification accuracy of many classic classification algorithms on medical data sets is not ideal.

Due to the high-dimensional feature space and high feature redundancy of medical data, it is necessary to perform feature selection operations when mining medical data. Feature selection technology can help people understand data, simplify machine learning and data mining models, reduce the computation time of training models, and maintain or improve the classification or prediction performance of models. Feature selection is broadly divided into two types: feature selection using the structure of the data itself and feature selection using external knowledge. There are many methods for feature selection based on the structure of the data itself, such as factor analysis, Relief-F [28], chi-square test, principal component analysis, and genetic algorithms. Biofilter is a method of feature selection using prior knowledge [29]. By adding biological knowledge to the model, the search space for variable selection can be significantly reduced. In general, any research question needs to be analyzed on a case-by-case basis. Whether using the data’s own structure or external knowledge, it is a good feature selection method if it effectively reduces the data dimensions and removes redundant information.

In order to predict the risk factors of IHD during pheochromocytoma surgery, firstly, the fast feature selection of Relief-F filtering was used to preprocess the data and filter out the important features. Then the classification effects of several machine learning algorithms were compared to find an algorithm suitable for analyzing the research data. Based on the characteristics of the data, the algorithm was further improved to obtain important factors that can be used to predict the occurrence of IHD.

## Methods

### Dataset

The study protocol was approved by the Institutional Research and Ethics Committee of Shengjing Hospital of China Medical University (No. 2019PS003K). Written informed consent was obtained from all patients. The clinical research registry unique identification number is ChiCTR1900020811. This study adheres to CONSORT guidelines.

The data set consists of 283 patient characteristics and 19 clinical parameters. The diagnosis of pheochromocytoma was confirmed by pathological examination, and patients who underwent either unilateral laparoscopic or open adrenalectomy were included. The clinical stage was localized (apparently benign) disease with an American Society of Anesthesiologists (ASA) score of 1–3. Patients were excluded if they had a familial history of pheochromocytoma, were converted to laparotomy, or underwent bilateral adrenalectomy or surgery for ectopic pheochromocytoma, see details in flowchart: supplementary Fig. 1.

The population information is in Table 1. The patients’ characteristics included sex, age, body mass index (BMI), and comorbidities: ASA score, diabetes mellitus, coronary heart disease (CHD), hypertension, and arrhythmia. The disease characteristics include tumor side and size, tumor necrosis, enhanced computed tomography difference. The preoperative parameters include the use of alpha adrenoreceptor antagonists, use of crystal/colloid fluids, preoperative transfusion, 24 h urine vanillylmandelic acid/upper normal limit value. The intraoperative parameters include surgical approach and IHD. There were 18 prediction indicators, and one target indicator, IHD occurrence. IHD was defined as the presence of at least one event of intraoperative systolic blood pressure > 200 mmHg and a mean arterial pressure < 60 mmHg, or the requirement for norepinephrine management or blood transfusion to maintain normal blood pressure intraoperatively [30]. Hypertension was classified into three categories: normal, intermittent, and continuous hypertension. Range of ASA score was 1–3.

**Table 1:** The population characteristics of patients

	Without IHD *n* = 209 (73.9)	With IHD *n* = 74 (26.1)	***p***-value
**Demographic characteristics**			
Mean age (years)	51.9 ± 12.3	54.0 ± 13.8	0.233
Sex (male/female)	110 (52.6) / 99 (47.4)	31 (41.9) / 43 (58.1)	0.112
BMI (kg/m^2^)	24.1 ± 3.5	21.9 ± 2.7	< 0.001
ASA score 1/2/3	52(24.9)/136(65.1)/21(10.0)	14(18.9)/53(71.6)/7(9.5)	0.548
**Comorbidity**			
Diabetes mellitus	61 (29.2)	23 (31.1)	0.759
Coronary heart disease	66 (31.6)	37 (50.0)	0.005
Hypertension Normal/Intermittent/Continuous	82(39.2)/47(22.5)/80(38.3)	30(40.5)/18(24.3)/26(35.1)	0.883
Arrhythmia	12 (5.7)	4 (5.4)	0.914^b^
**Preoperative data**			
Tumor side (left/right)	103 (49.3) / 106 (50.7)	39 (52.7) / 35 (47.3)	0.613
Radiographic tumor size (cm)	5.2 ± 2.5	6.5 ± 3.1	< 0.001
Tumor necrosis	69 (33.0)	33 (44.6)	0.075
Tumor enhanced CT difference (Hu)	43.2 ± 20.6	45.6 ± 20.2	0.435
Use of α adrenoreceptor antagonists	115 (55.0)	42 (56.8)	0.797
Use of crystal/colloid fluid	118 (56.5)	29 (39.2)	0.011
Use of blood transfusion	54 (25.8)	15 (20.3)	0.338
24-h urine metanephrines/ normal upper limit	1.4 (0.9–2.2)	1.47 (0.9–2.7)	0.153^a^
**Intraoperative data**			
Laparoscopic vs. Open	109 (52.2) / 100 (47.8)	42 (56.8) / 32 (43.2)	0.495

Continuous variables with normal distribution are reported as the mean ± standard deviation (SD), while non-normal continuous variables as the median (interquartile range) and categorical variables as numbers (percentages). Student’s *t*-test was used to compare the mean values of two continuous normally distributed variables and the Mann–Whitney U-test was used to determine mean values of two continuous non-normally distributed variables. The chi-squared or Fisher’s exact test was used for categorical variables

^a^ Mann–Whitney U-test

^b^ Fisher’s exact test

*BMI* body mass index; *ASA* American Society of Anesthesiologists; *CT* computed tomography; *IHD* intraoperative hemodynamic instability

Sex, CHD, arrhythmia, diabetes mellitus, tumor side, tumor necrosis, use of α adrenoreceptor antagonists, use of crystal/colloid fluid, use of blood transfusion, and surgery approach were regarded as categorical variables, all valued as 0 or 1. Hypertension was valued as 0, 1, or 2, whereas ASA score was valued as 1, 2, or 3; thus, these two variables were categorized as numerical variable and categorical variable, respectively. The computation was performed independently using seven models, and its purpose was to judge the target indicator (i.e., which indicators were closely related with the occurrence of IHD during surgery) by calculating the occurrence of all 18 indicators.

### Data mining

Relief-F is a feature selection algorithm with high operating efficiency. It expands the functions of multi-class data processing on the basis of the Relief algorithm and simultaneously solves the problem of noise and incompleteness of the data. The algorithm measures the importance of features by calculating a correlation statistic on each feature. The larger the correlation statistics of a feature, the more important this feature is in classification. By sorting all features and then setting a threshold or feature selection number, a filtered feature subset can be obtained. Relief-F was first used in this study to perform feature extraction on the dataset.

There are three main applications of machine learning algorithms: classification and regression, clustering, and dimension reduction. The clustering method has four applications: unsupervised medical image segmentation; studies of diseases subtype classification; relationship analysis and feature interpretation of disease and genes. Classification and regression can predict disease risk, postoperative recovery time prediction, surgical selection, and efficacy evaluation [31]. So, classification methods of data mining were used, including Naive Bayes, decision tree (CART, C4.5, C5.0, and C5.0 boosted), random forest algorithms and logistic regression. Naive Bayes algorithm is a classification method based on Bayes theorem, the characteristic conditional independence hypothesis, and a classification algorithm based on probability theory [32], This is a very powerful model for returning predicted values and certainty. This is easy to understand and implement [33]. It has also been used as a benchmark algorithm to compare other types of classification algorithms [34]. C4.5 is a decision tree algorithm [35] modified from the ID3 algorithm [36], and the gain ratio is used for disassociation in the Shannon entropy-based decision tree [37]. CART [38] is a decision tree algorithm that supports the splitting based on Gini value, binary system, and ordered binary system [39], and it realizes only binary splitting. C5.0 is a decision tree algorithm modified from theC4.5 algorithm. C5.0 boosted improves model accuracy. The main advantage of using decision trees is the visualization of data for classes. This visualization is useful because it makes it easy for users to understand the overall structure of the data, which property has the most impact on the class [34]. The random forest algorithm [40] is based on ensemble learning and a classifier containing multiple decision trees, and RF has proven to be a highly accurate algorithm in various fields including medical diagnostics [41]. The two most primitive and common methods for random forest used to measure the importance of features are MDA and MDG. The MDA measure converts the value of a variable into a random number, and random forest can predict the decline in accuracy. The larger a MDA value is, the more important the variable will be. The MDG measure calculates the influence of each variable on the heterogeneity of the observed values at each node of the classification tree using the Gini index, so as to compare the importance of variables; the larger the MDG value, the more important the variable will be. All analyses were performed using the Python and R 3.5.1 programming language, with the packages e1071, rpart, RWeka, C50, random forest, and caret.

The cross-validation method, hold-out method, and bootstrap method are used in the validation phase. The accuracy of each algorithm is calculated independently by dividing the train set and the test set. For the cross-validation method, the dataset is divided into *n* equal subsets, (*n*–1) subsets are used as train sets, one subset is used as the test set, and this process is repeated *n* times. In this study, the cross-validation was performed 5, 10, and 15-fold. For the hold-out method, the dataset is divided into one train set and one test set. In this study, the dataset was divided into two sets using three different divisions: 80% of the data for training and 20% of the data for testing; 70% of the data for training and 30% of the data for testing; and 60% of the data for training and 40% of the data for testing. For the bootstrap method, random samples are selected to create the test set and the train set. In this study, the test and train subsets contained 50, 100, or 200 samples.

### Model assessment

The model performance evaluation indicators (e.g., accuracy, error rate, sensitivity, and specificity) were calculated as predicted classes and actual classes in the confusion matrix. Samples with *y* = 0 were regarded as positive (normal patients) and those with *y* = 1 were regarded as negative (patients); *y* is the target variable used for classification.

Accuracy was calculated by dividing the number of records predicted correctly by the total number of samples in the confusion matrix:

$$ Accuracy=\frac{Number\ of\ records\ predicted\ correctly}{Total\ number\ of\ samples\ in\ the\ confusion\ matrix} $$

Other evaluation indicators were calculated according to the confusion matrix. The true positive rate (TPR) reflects model sensitivity (recall) and describes how many illness-free cases were recognized [i.e., the percentage of all recognized positive cases in all true positive (TP) cases and false negative (FN) cases, or TPR = TP/(TP + FN)]. The true negative rate (TNR) reflects model specificity and describes how many ill cases were recognized [i.e., the percentage of all recognized negative cases in all true negative (TN) cases and false positive (FP) cases, or TNR = TN/(FP + TN)]. The positive predictive value (PPV) reflects model precision and describes how many cases in the predicted illness-free cases were correct [PPV = TP/(TP + FP)]. Precision and recall generally had an inverse relationship [f1-score **=** 2 × recall × precision/(recall + precision)], where the f1-score integrated both precision and sensitivity (recall) and could be used as an evaluation indicator, f1-score is called F-Measure [42].

**Fig. 1:**
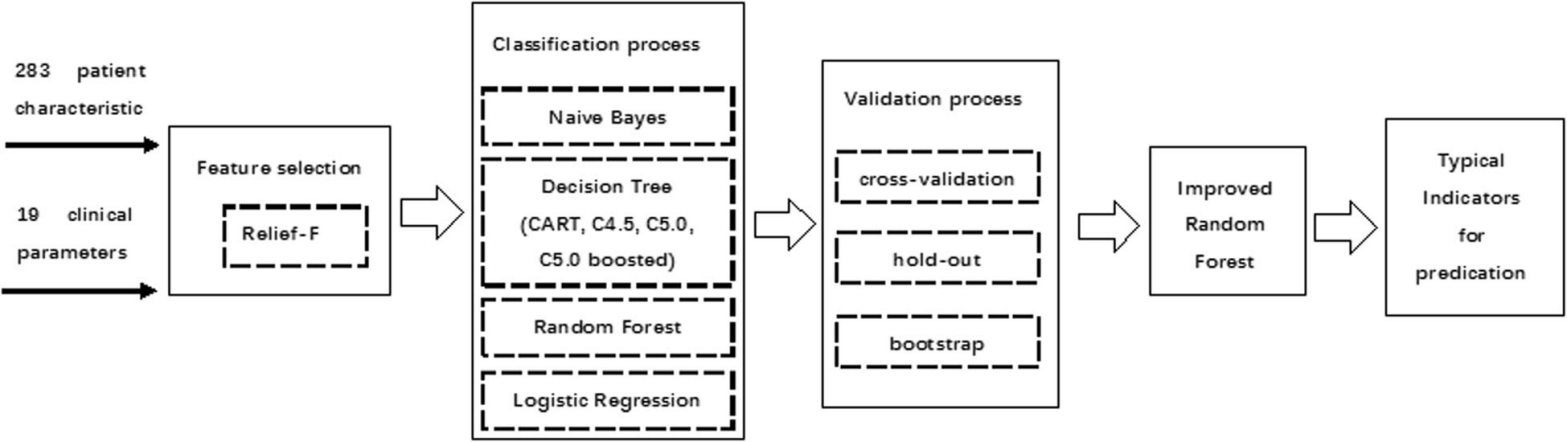
Flow chart for predicting IHD during pheochromocytoma surgery

Based on the accuracies of seven models obtained using different verification methods, the Receiver-Operating Characteristic (ROC) curve was plotted and then the area under ROC curve (AUC) was calculated to compare the classification effects of seven models. The ROC curve is termed a sensitivity curve, and is a comprehensive indicator reflecting the continuous variables of sensitivity and specificity. The ROC curve reveals the relationship between sensitivity and specificity by means of composition method, and supports the calculation of a series of sensitivities and specificities by setting several different critical values for the continuous variables. The AUC is generally 0.5–1.0; with larger AUC values indicating higher diagnostic accuracy. In the ROC curve, the points closest to the upper left of the coordinate system represent the critical values with high sensitivity and high specificity. The ROC curve is plotted using two variables, the *x*-coordinate is the false positive rate [FPR = FP/(FP + TN)] and the *y*-coordinate is TPR. Figure 1 shows the flow chart on predicting IHD during pheochromocytoma surgery.

## Results

The results of indicator weights using Relief-F are shown in Table 2. The larger the feature weight, the more important the feature is. The weights of eight indicators below − 24 were low and we considered them unimportant, and so excluded them. The remaining 10 indicators were used as the result of feature selection for further analysis. The following experiments were performed on the dataset after removing the unimportant feature variables.

**Table 2:** Indicator weights obtained by Relief-F

Attributes	Weights
ctvalue	−2.8871
prevma	−3.3417
arrhythmia	−4.4444
age	−4.6933
bmi	−6.7295
size	−9.2895
asa	−13.3596
preblood	−19.3992
hypertension	−22.7188
dm	−23.1212
**necrosis**	**−24.2869**
**surgeryapproach**	**−26.2391**
**preablock**	**−27.0017**
**chd**	**−27.0591**
**precrystal**	**−27.1358**
**precolloid**	**−27.1396**
**side**	**−27.5089**
**sex**	**−28.2274**

Seven models were applied 10 times respectively and their performance is shown in Table 3. The highest classification accuracy and AUC of the random forest model in the test set were achieved when hypertension and ASA were used as categorical variables and the training and test sets were divided using the hold-out method with a division ratio of 6:4. The AUC was 0.8636 and the calculated accuracy was 0.8509 from dividing the number of records predicted correctly (85 + 12) by the total number of samples in the confusion matrix (114) (Table 4). The next highest AUC and accuracy were 0.8630 and 0.8421, respectively, obtained with the random forest model in the numerical dataset. In addition, all values with accuracy > 0.8 were obtained by the random forest algorithm (Table 3), although the validation method and selection of the training and test sets differed. Therefore, when the hold-out method was used to divide the training and test sets, and the division ratio was 6:4, the random forest model had the highest classification accuracy on the test set.

**Table 3:** Accuracy and AUC values of all models

		hold out 80/20	hold out 70/30	hold out 60/40	CV 5 fold	CV 10 fold	CV 15 fold	bootstrap 50	bootstrap 100	bootstrap 200
Numerical IHD Dataset										
Logistic regression	Accuracy	0.7018	0.7529	0.7368	0.7386	0.7278	0.7207	0.6223	0.7486	0.7470
	AUC	0.5374	0.6392	0.6105	0.6096	0.5951	0.5597	0.5388	0.6343	0.6102
Naive Bayes	Accuracy	0.7544	0.7412	0.7719	0.7245	0.7319	0.7312	0.6524	0.7705	0.7590
	AUC	0.7392	0.7791	0.7999	0.6591	0.6740	0.7041	0.4966	0.6786	0.7719
CART	Accuracy	0.7719	0.7059	0.6930	0.7031	0.7036	0.7060	0.6567	0.7377	0.7349
	AUC	0.6827	0.3797	0.6999	0.6787	0.6097	0.6587	0.4495	0.6862	0.6694
C4.5	Accuracy	0.7193	0.7294	0.7544	0.7563	0.7284	0.7528	0.7167	0.7268	0.6747
	AUC	0.6520	0.6792	0.7569	0.6727	0.6991	0.7480	0.4422	0.6151	0.6233
C5.0	Accuracy	0.6667	0.6824	0.7544	0.7246	0.7318	0.7493	0.6652	0.7268	0.6747
	AUC	0.6478	0.7132	0.7716	0.6514	0.7150	0.7146	0.4847	0.3861	0.6498
C5.0 boosted	Accuracy	0.7018	0.7882	0.7544	0.7706	0.7499	0.7596	0.6695	0.7268	0.7590
	AUC	0.6420	0.7710	0.7686	0.7415	0.7130	0.7510	0.6600	0.6988	0.7849
Random Forest	Accuracy	0.7544	0.7765	**0.8421**	0.8023	0.8025	0.8123	0.7639	0.8033	0.7952
	AUC	0.8181	0.8524	**0.8630**	0.7943	0.8268	0.8274	0.6923	0.8538	0.8533
Categrical IHD dataset										
Logistic regression	Accuracy	0.6842	0.7411	0.7544	0.7563	0.7493	0.7483	0.5794	0.7377	0.7349
	AUC	0.5257	0.6448	0.6220	0.6255	0.6442	0.6322	0.5535	0.6191	0.6179
Naive Bayes	Accuracy	0.7544	0.7412	0.7632	0.7245	0.7319	0.7312	0.6481	0.7650	0.7590
	AUC	0.7359	0.7812	0. 7986	0.6565	0.6745	0.7012	0.4976	0.6580	0.7711
CART	Accuracy	0.7368	0.7059	0.6930	0.7031	0.7108	0.7097	0.6567	0.7377	0.7349
	AUC	0.6653	0.3797	0. 6999	0.6787	0.5971	0.6575	0.4495	0.6862	0.6694
C4.5	Accuracy	0.7193	0.7412	0.7632	0.7456	0.7461	0.7774	0.7554	0.6831	0.7108
	AUC	0.4427	0.7037	0. 7580	0.6784	0.6818	0.7365	0.4641	0.5457	0.6575
C5.0	Accuracy	0.7193	0.6706	0.7544	0.7316	0.7459	0.7528	0.6395	0.7268	0.6747
	AUC	0.6171	0.6939	0. 7716	0.6775	0.6983	0.6994	0.6209	0.3861	0.6701
C5.0 boosted	Accuracy	0.7544	0.7529	0.7719	0.7598	0.7562	0.7943	0.6395	0.7541	0.7470
	AUC	0.7575	0.7283	0. 7084	0.7318	0.7335	0.7947	0.6209	0.7169	0.7596
Random Forest	Accuracy	0.7719	0.7765	**0.8509**	0.8093	0.8130	0.8123	0.7811	0.8197	0.7952
	AUC	0.8198	0.8597	**0. 8636**	0.7782	0.8194	0.8179	0.7064	0.8542	0.8322

**Table 4:** The confusion matrix of the random forest model

		Actual classes	
		Positive	Negative
Predicted classes	Positive	85 (True Positive, TP)	2 (False Positive, FP)
	Negative	15 (False Negative, FN)	12 (True Negative, TN)

After feature extraction, there were 283 samples in the data set, there were 10 attributes, 74 positive samples, and 209 negative samples, and the ratio of the number of positive to negative samples was 1:2.82. For continuous variables, we tried to use the improved random forest algorithm to further get the indicators to predict IHD during pheochromocytoma surgery.

Imbalanced Data Random Forest (BRF) algorithm idea:

Before training the random forest classifier, the algorithm first uses the bootstrap method to randomly extract a consistent number of sample subsets from the majority class sample set and the minority class sample set, and then extract the majority class sample subset. Then this is recombined with a small number of sample subsets to obtain a balanced training data set with sample category distributions, and then a random forest classifier is trained on this balanced training set to form a “forest” of random forests. When an unknown sample is classified or predicted, the category of the sample is determined by voting from multiple random forest classifiers. The proposed algorithm flow is explained as follows:

- Input: Dataset *D*, Number of RF-based classifiers in BRF
- Output: Random Forest Classifier for Imbalanced Data *BRF*(*x*)

Stages:

1. Set m is the number of RF-based classifiers and n is the sample number randomly sampled;
2. Divide the training set D into a subset of majority samples *D*_m*ajority*_ and subset of minority *D*_min*ortity*_;

(for) *i* = 1, 2, ⋯, *m*

1. Resample *D*_m*ajority*_ randomly with replacement, get ${D}_{\mathrm{m} ajority}^{sampling}$ , set $\left|{D}_{\mathrm{m} ajority}^{sampling}\right|=n$;
2. Random resampling *D*_min*ority*_ with replacement, get ${D}_{\min ority}^{sampling}$ , set $\left|{D}_{\min ority}^{sampling}\right|=n$;
3. Generate training data, set ${D}_{train}=\left|{D}_{\mathrm{m} ajority}^{sampling}\right|+\left|{D}_{\min ority}^{sampling}\right|$;
4. Generate test data, set *D*_*test*_ = *D* − *D*_*train*_;
5. Train random forest classifier *RF*_*i*_(*x*) on *D*_*train*_;

and for

3. $$ BRF(x)=\operatorname{sgn}\sum \limits_{i=1}^m{RF}_i(x) $$;
4. (Output)*BRF*(*x*)

**Fig. 2:**
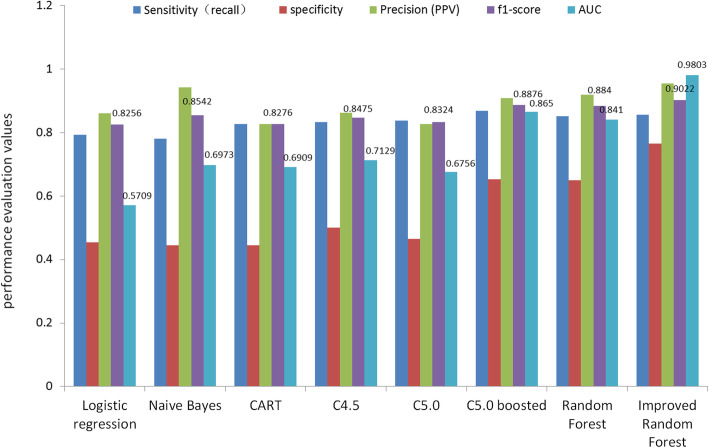
Comparison of multiple evaluation indicators

**Fig. 3:**
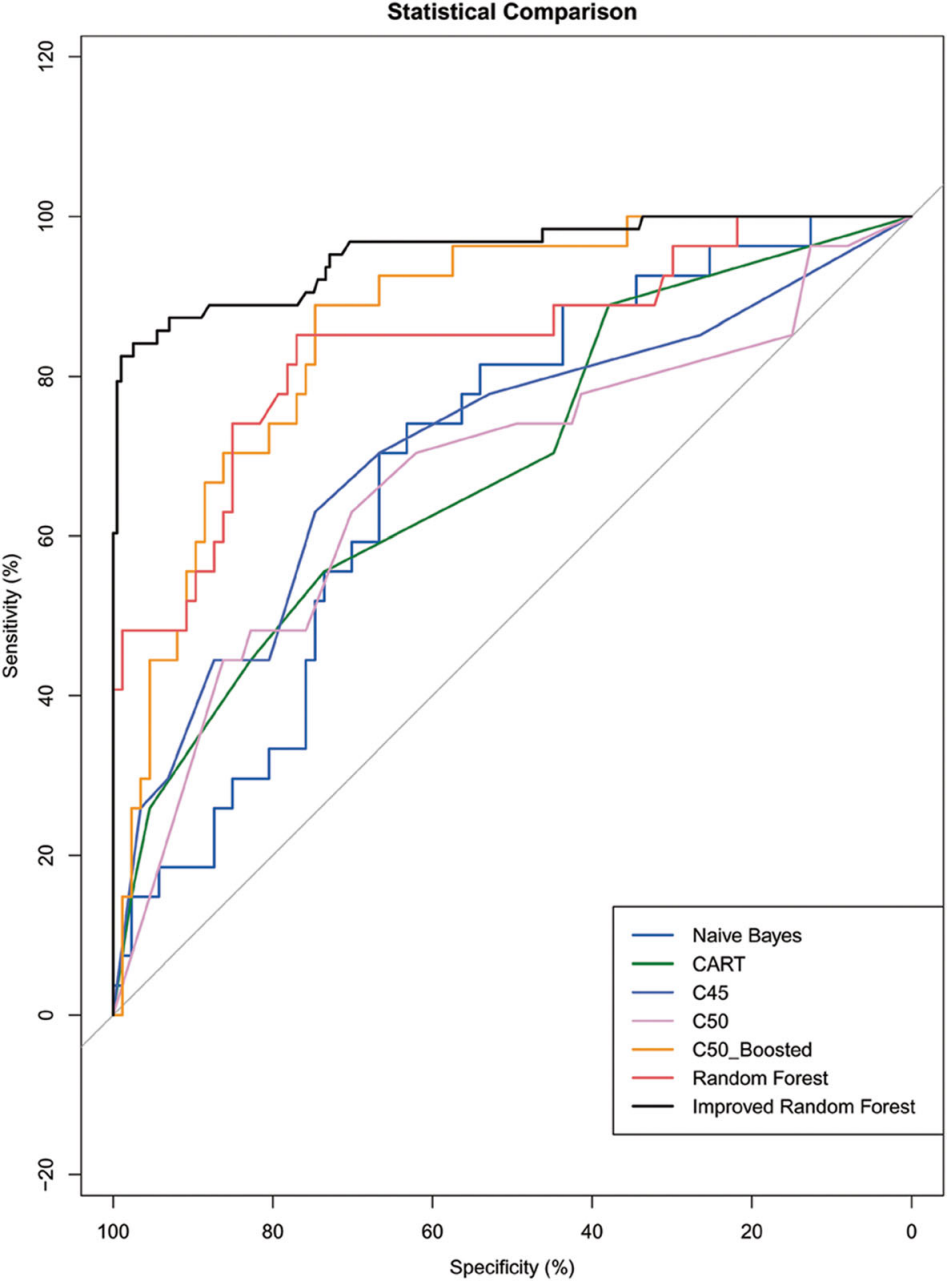
Receiver-Operating Characteristic curve for prediction of hemodynamics instability

Considering all attributes as continuous variables, the improved random forest algorithm was compared with other algorithms. Other algorithms took 60% of the data set as the training set and the rest as the test set. The experimental results are shown in Fig. 2. The comparison results between the improved random forest algorithm ROC curve and other algorithms are shown in Fig. 3. We compared the results for AUC, sensitivity (recall), specificity, precision, and f1-score among the improved random forest and other models, the improved random forest model had the highest AUC (0.9803), specificity (0.7647), and precision (0.954) among all algorithms, including relatively higher sensitivity (recall) (0.8557) and highest f1-score (0.9022) integrating recall and precision. The 95% confidence interval from random forests is (0.772, 0.9107). A Confidence Interval is a range of values we are fairly sure our true value lies in. For the same sample estimate for the same population, the 99% confidence interval has higher credibility, and its true value has higher credibility, but its interval width is large and inaccurate; the 95% confidence interval is less reliable than the 99% interval, but its accuracy is higher. The choice of 95% confidence is very common in presenting confidence intervals, although other less common values are used, such as 90 and 99.7%. In practice, we can use any value you prefer [43, 44]. The comparative analysis showed that the random forest model had optimal classification performance.

**Fig. 4:**
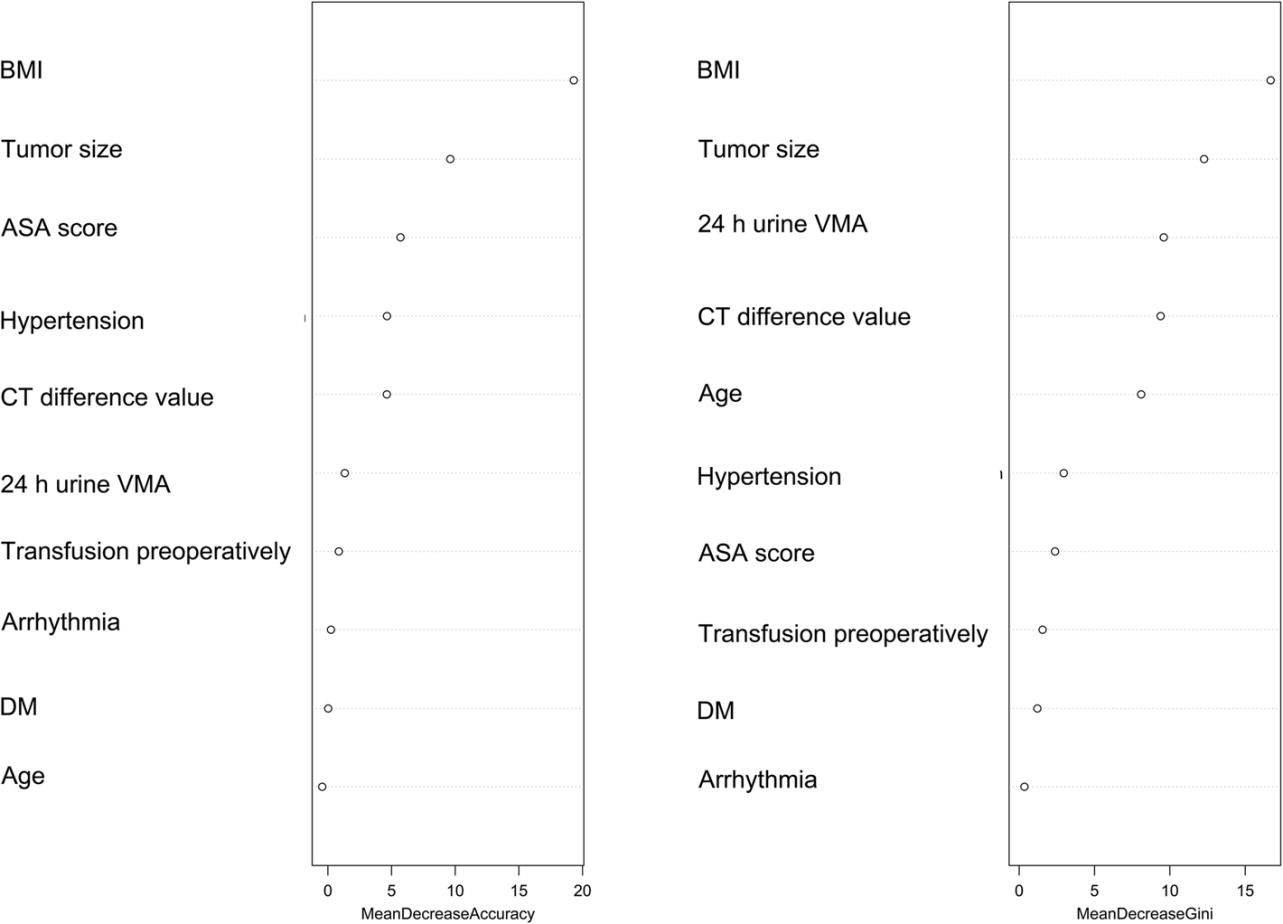
Visualization of important attribute scores

An illustrative diagram of important attribute scores is presented in Fig. 4 and Table 5. The important attribute scores calculated using the Mean Decrease Accuracy (MDA) were BMI, tumor size, ASA, hypertension, and enhanced computed tomography difference – the values exceeded 4.6. Those calculated using the Mean Decrease Gini (MDG) were BMI, tumor size, 24-h urine vanillylmandelic acid/upper normal limit value, enhanced computed tomography difference, and mean age – the values exceeded 8. The weights of attributes from Relief-F were enhanced computed tomography difference,24-h urine vanillylmandelic acid/upper normal limit value, arrhythmia, mean age, and BMI – the values exceeded − 6.8. We chose the indicators that appeared in more than two permutations as the final predictors. Thus, BMI, tumor size, 24-h urine vanillylmandelic acid/upper normal limit value, enhanced computed tomography difference, and mean age could be used as risk factors for predicting IHD during pheochromocytoma surgery.

**Table 5:** Important attribute scores according to the improved random forest model

Attributes	Importance Scores of random forest	Attributes	Importance Scores of random forest	Attributes	Relief-F
	Mean Decrease Accuracy		Mean Decrease Gini		Weight of attribute
bmi	19.3095	bmi	16.688	ctvalue	−2.8871
size	9.6143	size	12.2722	prevma	−3.3417
asa	5.7061	prevma	9.5934	arrhythmia	−4.4444
hypertension	4.6416	ctvalue	9.3884	age	−4.6933
ctvalue	4.6293	age	8.0945	bmi	−6.7295
prevma	1.3311	hypertension	2.9614	size	−9.2895
preblood	0.8616	asa	2.3838	asa	−13.3596
arrhythmia	0.2419	preblood	1.5554	preblood	−19.3992
dm	0.0276	dm	1.2103	hypertension	−22.7188
age	−0.4422	arrhythmia	0.3524	dm	−23.1212

## Discussion

This study aimed to predict the risk factors of IHD during pheochromocytoma surgery by data mining. With the necessary feature extraction steps, the biomedical data with small samples and high dimensions were analyzed, and the Relief-F algorithm used to eliminate irrelevant features and screen features conducive to the prediction of minority classes. The results showed that random forest had the highest accuracy (0.8509) and was the best classification model among the seven data mining models. Then, due to the imbalance of the data, we improved the random forest algorithm to obtain the best classification performance.

In related fields such as medical diagnosis and biomedical data analysis, imbalanced data are more common. It is difficult to analyze and mine this kind of data with traditional methods, and it causes problems such as overfitting and dimensional disaster. High-dimensional data contain a large number of unrelated redundant features. A classification model constructed using the original data set will reduce the prediction effect and interpretability. Research shows that using feature selection alone can solve the problem of high-dimensional imbalanced data classification, and it is more helpful to improve performance than classification algorithms. The idea of introducing an imbalanced classification method and determining the most relevant influencing factors for disease classification with the least redundancy are of great significance for disease prevention diagnosis and drug development screening.

Random forest, developed by Leo Breiman and Adele Culter in 1999, is a classification algorithm composed of a multitude of decision trees [31]. Many studies have shown that the random forest algorithm has high accuracy in various fields including medicine. For example, in HIV/AIDS medicine study, compared with the J48 algorithm and neural network, random forest predicted virus response with comparable accuracy [45]. In a landslide susceptibility assessment, random forest had the best AUC and accuracy value compared with best-first decision tree and Naive Bayes [46].

In order to assess which variables are important within the random forest algorithm, two measures can be used: MDA and MDG [47–51]. The latter is based on the number of splits within the decision trees for each predictor, and is criticized for its bias for continuous variables. In the random forest algorithm, there are more options for analyses of continuous variables regarding where splits can occur within each decision tree, and the MDG value tends to give higher importance to these variables compared to ordinal or categorical variables, which have a limited number of places for splits to occur. Another important measure is the MDA, which is the difference between the out-of-bag error rate from a randomly permuted dataset and the out-of-bag error rate of the original dataset, expressed as an average percent over all trees in the forest. For both of these important measures, high values represent important variables and low values represent unimportant variables within the random forest framework.

In the application for analyzing clinical data, data mining can help find potential relationships between many clinical manifestations and diseases. Meanwhile, clinicians are also interested in the predictors of many diseases. In this study, the important attributes were BMI, mean age, 24 h urine vanillylmandelic acid/upper normal limit value, tumor size, enhanced computed tomography difference. BMI was an independent risk factor for both severe and cardiovascular morbidity, and was reported previously as a risk factor for IHD [52, 53]. Lower BMI is associated with less effective circulatory volume due to relatively lower body weight, resulting in large fluctuations in blood pressure and a high incidence of IHD. Currently, there is only one study that investigated the intraoperative changes in hemodynamics in a Chinese population with pheochromocytoma; the results show an association between age > 45 years and IHD [1], consistent with our study results. The final metabolite of CA is vanillylmandelic acid, so the 24-h urine vanillylmandelic acid/upper normal limit value is an important factor influencing the occurrence of IHD and a biochemical indicator with clinical importance.

Tumor size was also an effective predictor for IHD in our study, in agreement with the reports of previous studies [24, 54]. A large pheochromocytoma has a more prominent network of vessels and is associated with greater intraoperative blood loss than smaller tumors [55, 56]. Large tumors secrete higher levels of CAs, which can easily to lead to greater fluctuations in blood pressure during the perioperative period [57]. Natkaniec et al. [58] reported that intraoperative blood loss in 530 patients who underwent laparoscopic adrenalectomy was significantly greater in patients with tumor diameters ≥6 cm than those with diameters < 6 cm.

Usually, pheochromocytoma patients have a higher incidence of heart disease than those with essential hypertension [53]. Because the myocardium and coronary arteries are exposed to abnormally elevated levels of CAs for prolonged periods, this can lead to collagen deposition and fibrosis formation in the myocardium. However, this factor was not included in this study.

Other effective predictors for IHD involvement are the use of α adrenoreceptor antagonists, crystal/colloid, and blood transfusion for volume expansion before surgery. It was confirmed that PMP is important to decrease fluctuations in blood pressure during the perioperative period [59, 60]. All patients with pheochromocytoma should receive PMP and volume expansion to block the effects of released CAs [61]. However, these factors were not included in our study. This may be due to the relatively small sample size and number of events included in this study, and may lead to underestimation of its predictive effect. Nevertheless, both PMP and volume expansion are very important to achieve a good treatment outcome.

There were several limitations to this study. First, some variables related to IHD were not considered, such as patient symptoms, genomic characteristics, and the dosage and duration of preoperative medical preparations. Second, the random forest program does not generate traditional statistical measurement values (e.g., *p* value and test statistics). There are many alternative protocols to obtain these statistical data, but it may be challenging to implement a completely different analysis framework. For example, the random forest algorithm provides two measures for the importance of variables, and it may be beneficial or not for the predictor depending on the measurement scale or the number of categorical variable sets. The measurement of importance is criticized because the above-mentioned variables are highly sensitive to the number of trees in the forest and the number of selected prediction variables, and they are both user-defined parameters. The importance of variables is not necessarily identical to the statistical significance. A variable may be very important in the random forest model but not statistically or clinically significant. Some investigators have proposed a method to test the statistical significance of variables in the random forest framework, but there is no direct and recognized approach for such an application [45, 62, 63].

## Conclusions

Surgery for pheochromocytoma may induce excessive release of CA into the blood circulation, thereby producing a high risk for IHD and increased mortality. This study analyzed clinical data of 283 patients with pheochromocytoma surgery using feature selection and a classification method for imbalanced data, and determined the optimal model for predicting IHD during pheochromocytoma surgery. The improved random forest model had the best AUC and accuracy among all tested models. The BMI, mean age, 24 h urine vanillylmandelic acid/upper normal limit value, tumor size, and enhanced computed tomography difference were important indicators predicting occurrence of IHD during pheochromocytoma surgery.

The current trends of increasing use of electronic medical records and generating ever increasing volumes of medical data ensure that data mining technologies will be increasingly applied in clinical and medical decision-making, and provide continually expanding support for the diagnosis, treatment, and prevention of various diseases.

## Supplementary information

**Additional file 1 Supplementary figure 1.** Flowchart.

**Additional file 2.** CONSORT Checklist of items to include when reporting a randomized trial.

## Data Availability

The data generated and analyzed during this study cannot be made publicly available due to regulations at the institutional review board concerning the potential of disclosure of an individual’s personal health information. Please contact the corresponding author regarding access to anonymized data.

## Abbreviations

IHD: Intraoperative hemodynamic instability
AUC: Area under curve
CA: Catecholamine
PMP: Preoperative medical preparation
BMI: Body mass index
CHD: Coronary heart disease
TPR: True positive rate
TP: True positive
FN: False negative
TNR: True negative rate
TN: True negative
FP: False positive
ROC: Receiver-Operating Characteristic
BRF: Imbalanced Data Random Forest
MDA: Mean Decrease Accuracy
MDG: Mean Decrease Gini
