# Supplementary figures and images for: Application of data mining for predicting hemodynamics instability during pheochromocytoma surgery
Source: BMC Med Inform Decis Mak. 2020 Jul 20;20:165. doi: 10.1186/s12911-020-01180-4 (PMC7370474; doi:10.1186/s12911-020-01180-4)

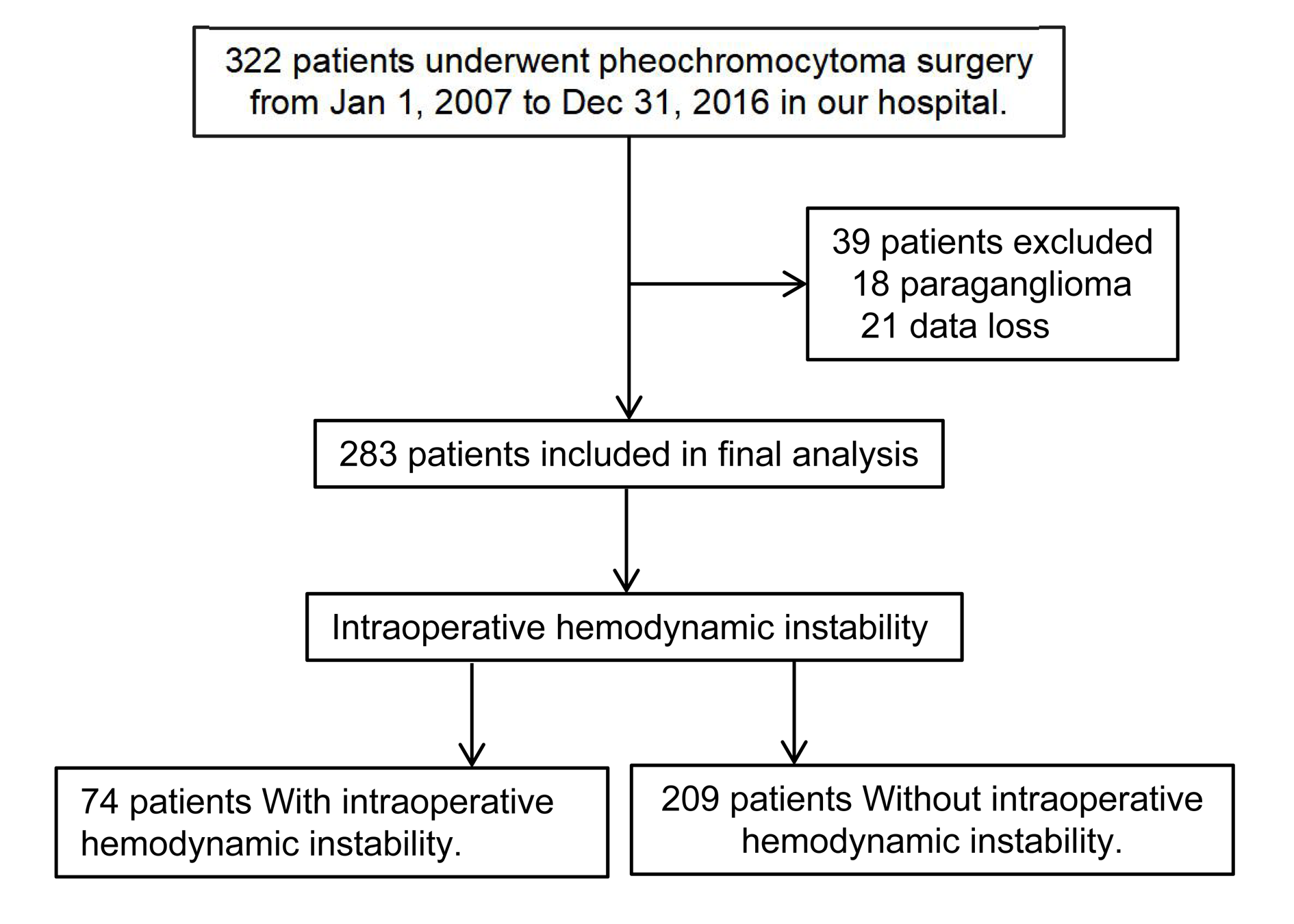

Supplement: Supplementary file 1 — Additional file 1 Supplementary figure 1. Flowchart. [file 12911_2020_1180_MOESM1_ESM.tif]
